# Effects of model size and composition on quality of head‐and‐neck knowledge‐based plans

**DOI:** 10.1002/acm2.14168

**Published:** 2023-10-05

**Authors:** Robert Kaderka, Nesrin Dogan, William Jin, Elizabeth Bossart

**Affiliations:** ^1^ Department of Radiation Oncology University of Miami Miller School of Medicine Miami Florida USA

**Keywords:** automated planning, head‐and‐neck, knowledge‐based planning

## Abstract

**Purpose:**

Knowledge‐based planning (KBP) aims to automate and standardize treatment planning. New KBP users are faced with many questions: How much does model size matter, and are multiple models needed to accommodate specific physician preferences? In this study, six head‐and‐neck KBP models were trained to address these questions.

**Methods:**

The six models differed in training size and plan composition: The *KBP_Full_* (*n* = 203 plans), *KBP_101_* (*n* = 101), *KBP_50_* (*n* = 50), and *KBP_25_* (*n* = 25) were trained with plans from two head‐and‐neck physicians. *KBP_A_* and *KBP_B_* each contained *n* = 101 plans from only one physician, respectively. An independent set of 39 patients treated to 6000–7000 cGy by a third physician was re‐planned with all KBP models for validation. Standard head‐and‐neck dosimetric parameters were used to compare resulting plans. *KBP_Full_* plans were compared to the clinical plans to evaluate overall model quality. Additionally, clinical and *KBP_Full_* plans were presented to another physician for blind review. Dosimetric comparison of *KBP_Full_* against *KBP_101_*, *KBP_50_*, and *KBP_25_* investigated the effect of model size. Finally, *KBP_A_* versus *KBP_B_* tested whether training KBP models on plans from one physician only influences the resulting output. Dosimetric differences were tested for significance using a paired *t*‐test (*p* < 0.05).

**Results:**

Compared to manual plans, *KBP_Full_* significantly increased PTV Low D95% and left parotid mean dose but decreased dose cochlea, constrictors, and larynx. The physician preferred the *KBP_Full_* plan over the manual plan in 20/39 cases. Dosimetric differences between *KBP_Full_*, *KBP_101_*, *KBP_50_*, and *KBP_25_* plans did not exceed 187 cGy on aggregate, except for the cochlea. Further, average differences between *KBP_A_* and *KBP_B_* were below 110 cGy.

**Conclusions:**

Overall, all models were shown to produce high‐quality plans. Differences between model outputs were small compared to the prescription. This indicates only small improvements when increasing model size and minimal influence of the physician when choosing treatment plans for training head‐and‐neck KBP models.

## 1 INTRODUCTION

Treatment planning in radiation therapy is time‐consuming and has been reported to be highly variable between planners.^1^ This variability can reduce plan quality and thus may lead to suboptimal outcomes for patients.^2^, ^3^, ^4^ Knowledge‐based planning (KBP) has been proposed as an automated tool to reduce planning variability, improve plan quality, and decrease planning time. With tools from machine learning, KBP utilizes sets of previously treated (or, sometimes, re‐optimized) plans to predict achievable dose‐volume histograms (DVH) for new patient plans.^5^, ^6^, ^7^, ^8^, ^9^, ^10^, ^11^, ^12^ These predictions can then be used to automatically generate optimization objectives, decreasing the amount of trial and error needed in manual treatment planning. Several clinics that utilize KBP demonstrated that KBP plans were non‐inferior to manual planning and that planning time could be decreased.^13^, ^14^, ^15^, ^16^

To create high‐quality plans using a KBP model, a database of “good” plans is required to train the model. Quality filtering is suggested to improve trained models.^5^, ^6^, ^7^, ^17^, ^18^ In this process, after initial training the DVH estimations for each plan in the training sets are evaluated. If a specific organ in a plan exceeds the dose predicted, one can eliminate this instance of the organ from the training set. The entire model is then retrained. By removing this outlier, a more accurate DVH prediction is then achieved. Further, the choice of optimization objectives has been shown to impact the quality of resulting plans and therefore, it is recommended to carefully fine‐tune optimization objectives.^14^ As a result of these considerations, it takes quite a few resources for a clinic to build their own KBP model. Clinics looking to build their own models are faced with many questions: Does model size influence resultant plan quality? Do planning preferences of different treating physicians influence the resulting plans from a KBP model? Will a model be applicable for a new or different physician? How many plans are needed to make a reasonable model?

It can be challenging if a clinic does not have access to copious amounts of previously treated plans, if the personnel time is limited, or if there is not the local expertise to tune such a model. Given this, there is an interest in the applicability of KBP models shared across institutions.^19^, ^20^, ^21^, ^22^, ^23^, ^24^ Institutional or physician bias can affect choices in what is considered when making planning decisions for patients.^25^ Several questions arise for a clinic such as whether an outside model can match institutional or physician standards for treatment, whether such physician preferences affect the model built, or if a model appeases each physician's preferences. While these questions have partially been answered in a prostate model,^26^ the applicability of the conclusions to more complex disease sites has yet to be tested. This study aims to shed light on these questions for head‐and‐neck (HN), which represents one of the most complicated disease sites treated with multiple targets at different dose levels, complex target shapes and proximity of many critical organs‐at‐risk (OAR).

In particular, this study seeks to answer the questions of whether increasing model size necessarily improves plan quality, and whether each physician requires their own KBP model to meet their planning preferences. Six HN KBP models which included volumetric modulated arc‐therapy (VMAT) plans were built for this purpose and tested by re‐optimizing an independent test set of VMAT plans. Models were built with differences in model size and different physician plan composition to evaluate the effect of size and potential differences when using specific patient cohorts for training.

## 2 METHODS

### 2.1 KBP training

Models were built in a commercial KBP solution (RapidPlan, ver. 16.1, Varian Medical Systems, Palo Alto, California). The makeup of the models is listed in Table 1. The “*KBP_Full_*” model was trained with 203 HN patients treated at our institution with VMAT between 2013 and 2019. Patients were treated with simultaneous integrated boost with 2−3 target levels with the Planning Target Volume (PTV) High receiving 6000−7000 cGy in 30−35 fractions. The “*KBP_101_*”, “*KBP_50_*”, and “*KBP_25_*” models were trained selecting 101, 50, and 25 out of these 203 patients respectively, approximately half of which were from each physician “A” and physician “B”. The “*KBP_A_*” and “*KBP_B_*” models were trained with 101 plans each, but exclusively consisted of patients treated by physician A and physician B, respectively. Where possible the models were matched to have similar makeup in terms of diagnosis (see Table 1). Prescriptions by physicians A and B were evaluated and it was found that physician A had stricter limits on cord Dmax by 700 cGy. Physician B had stricter constraints on brainstem, optics, oral cavity, constrictors, submandibulars and lips by 500−1500 cGy. Quality filtering was performed on the models as described in the introduction.

**TABLE 1:** Plan composition.

Diagnosis	*KBP_Full_*	*KBP_101_*	*KBP_50_*	*KBP_25_*	*KBP_A_*	*KBP_B_*	*Test set*
Thyroid	7	4	2	1	3	4	0
Glottis	20	10	5	3	9	11	5
Tonsil	69	31	15	7	34	35	5
Tongue	60	29	14	6	30	30	5
Parotid	7	6	3	2	4	3	5
Oropharynx	14	6	3	2	6	8	5
Hypopharynx	10	5	2	1	5	5	5
Uvula	1	1	1	1	1	0	0
Larynx	12	6	3	1	8	4	5
Floor of mouth	2	2	1	1	1	1	0
Pyriform sinus	1	1	1	0	0	1	0
Maxillary sinus	0	0	0	0	0	0	2
Nasopharynx	0	0	0	0	0	0	2
Total cases	203	101	50	25	101	101	39

*Note*: Details on the diagnosis and composition of the KBP models and test cases.

As shown in previous literature, optimization objectives strongly influence resulting plans.^14^ In this study, the aim was to determine plan differences based on the training set and not optimization objectives. Therefore, one set of optimization objectives was developed and fine‐tuned using the *KBP_Full_* model: 39 independent test plans were re‐optimized using the initial optimization objectives of the *KBP_Full_* model. To not bias the results, the 39 test patients were taken from a cohort of patients treated by a third head‐and‐neck physician at our institution. Plan differences in the clinical plans were compared in terms of DVH parameters for targets and OARs that are typically evaluated in HN treatment planning. Aggregate differences of KBP in clinical plans were analyzed and used to adjust KBP optimization objectives in an iterative manner. The final optimization objectives were then applied to all KBP models (*KBP_Full_*, *KBP_101_*, *KBP_50_, KBP_25_*, *KBP_A_*, *KBP_B_)* and are shown in Table 2. In other words, optimization objectives between the KBP models were identical except for the generated line objectives. This study aims at investigating differences in resulting plans based on changes in the training sets only. By keeping optimization objectives the same—except for the line objectives which are generated based on the training data set—any differences in resulting plans from the models are thus solely due to differences in the underlying training set.

**TABLE 2:** Optimization objectives.

Parameter	Type	Vol	Dose	Priority
PTV high	Upper	0%	103%	175
	Upper	0%	100%	0
	Lower	100%	99	175
	Lower	98%	100%	175
PTV intermediate high	Upper	0%	103%	150
	Upper	0%	100%	0
	Lower	100%	99%	200
	Lower	98%	100%	200
PTV intermediate	Upper	0%	103%	150
	Upper	0%	100%	0
	Lower	100%	99%	200
	Lower	98%	100%	200
PTV low	Upper	0%	103%	150
	Upper	0%	100%	0
	Lower	100%	99%	200
	Lower	98%	100%	200
BrachialPlexus_L	Upper	0%	6000cGy	100
BrachialPlexus_R	Upper	0%	6000cGy	100
Brainstem	Upper	0%	68%	200
	Upper	0%	54%	100
	Line	Generated	Generated	50
Chiasm	Upper	0%	4500cGy	200
	Line	Generated	Generated	30
Cochlea Lt	Line	Generated	Generated	100
Cochlea Rt	Line	Generated	Generated	100
Esophagus	Line	Generated	Generated	125
Eye Lt	Upper	0%	4500cGy	125
	Line	Generated	Generated	50
Eye Rt	Upper	0%	4500cGy	125
	Line	Generated	Generated	50
Larynx	Line	Generated	Generated	125
Lens L	Upper	0%	900cGy	125
	Line	Generated	Generated	30
Lens R	Upper	0%	900cGy	125
	Line	Generated	Generated	30
Lips	Line	Generated	Generated	60
Mandible	Upper	0%	100%	50
	Line	Generated	Generated	40
OpticNerve_L	Upper	0%	4500cGy	200
	Line	Generated	Generated	30
OpticNerve_R	Upper	0%	4500cGy	200
	Line	Generated	Generated	30
OralCavity	Upper	50%	3200cGy	75
	Line	Generated	Generated	50
Pacemaker	Upper	0%	200cGy	100
Parotid_L	Upper	50%	1600cGy	150
	Mean		2400cGy	150
	Line	Generated	Generated	50
Parotid_R	Upper	50%	1600cGy	150
	Mean		2400cGy	150
	Line	Generated	Generated	50
SpinalCord	Upper	0%	59%	200
	Upper	0%	48%	100
	Line	Generated	Generated	50
SpinalCord + 3 mm	Upper	0%	68%	200
	Line	Generated	Generated	50
Submandibular_L	Mean		3400cGy	60
	Line	Generated	Generated	60
Submandibular_R	Mean		3400cGy	60
	Line	Generated	Generated	60
Thyroid	Mean		63%	50
Normal tissue objective	Distance from target border: 0.20 cm			120
	Start dose: 100%			
	End dose: 30%			
	Fall‐off: 0.2 cm			

*Note*: These optimization objectives were set for all six KBP models. Therefore, the only changes in optimization objectives were due to the generated line objectives.

### 2.2 Comparison of plans

To analyze the differences resulting from the different training sets, the 39 test patients from a third physician were then re‐planned using each of the six KBP models. All KBP plans were normalized to the same V100% for the PTV High of the clinical plan (typically, but not always, V100% = 95%) for comparability. Dosimetric parameters were obtained for Body, PTV High/Int/Low, brainstem, cochlea, constrictors, cord, eyes, mandible, larynx, optic chiasm, optic nerves, oral cavity, parotids, and submandibular glands. For further plan evaluation, conformity index (CI) (prescription volume/target volume),^27^ homogeneity index (HI) ((D2%–D98%)/D50%)^28^ and body V100%, V50%, V20%, and V5% were calculated. These parameters were compared for clinical plans and *KBP_Full_* plans to evaluate the overall dosimetric quality of the KBP model. Differences in the mean parameters averaged over all 39 plans were tested for significance using a paired *t*‐test (*p* < 0.05). Differences in variance between the sets of plans were tested for significance using the *F*‐test (*p* < 0.05).

Clinical and *KBP_Full_* plans were also evaluated by a fourth independent physician. Both sets of plans were randomly blinded to plan 1 and plan 2. The physician was asked to evaluate whether plans were clinically acceptable, and which plan they preferred. If possible, the physician was asked to provide brief reasoning for their choice.

To analyze the effects of increasing KBP model size, a dosimetric analysis was then performed for *KBP_Full_* versus *KBP_101_*_,_ *KBP_50_*, and *KBP_25_*. Finally, the influence of physician preferences when building KBP models was investigated by a dosimetric comparison of *KBP_A_* to *KBP_B_* generated plans. As described above, dosimetric differences were tested for significance using a paired *t*‐test and *F*‐test (*p* < 0.05).

## 3 RESULTS

An overview of all dosimetric results is given in Tables 3 and 4. Table 3 gives the average value and standard deviation for all dosimetric parameters across the entire patient cohort for the clinical plans, *KBP_Full_*, *KBP_101_*_,_ *KBP_50_*, *KBP_25_*, *KBP_A_*, and *KBP_B_* plans. Table 4 gives the difference in the average DVH parameters of the clinical plans and *KBP_Full_*, *KBP_Full_* to *KBP_101_*_,_ *KBP_50_*, and *KBP_25_* as well as the difference of *KBP_A_* and *KBP_B_*. Table 4 also gives the standard deviation of differences and highlights if differences were statistically significant.

**TABLE 3:** Dosimetric parameters of all plans.

Parameter	*n*	Clinical	*KBP_Full_*	*KBP_101_*	*KBP_50_*	*KBP_25_*	*KBP_A_*	*KBP_B_*
Conformity Index	39	1.090 ± 0.076	1.054 ± 0.065	1.057 ± 0.065	1.049 ± 0.066	1.050 ± 0.067	1.053 ± 0.025	1.057 ± 0.066
Homogeneity Index	39	0.073 ± 0.039	0.077 ± 0.025	0.078 ± 0.025	0.075 ± 0.024	0.076 ± 0.025	0.077 ± 0.025	0.078 ± 0.026
Body V100% [%]	39	1.102 ± 0.809	1.061 ± 0.777	1.064 ± 0.781	1.056 ± 0.777	1.058 ± 0.781	1.081 ± 0.775	1.065 ± 0.782
Body V50% [%]	39	10.37 ± 3.33	9.45 ± 2.91	9.48 ± 2.91	9.45 ± 2.84	9.37 ± 2.81	9.19 ± 2.83	9.44 ± 2.91
Body V20% [%]	39	25.08 ± 5.70	24.42 ± 5.61	24.51 ± 5.62	24.42 ± 5.64	24.38 ± 5.54	24.44 ± 5.60	24.50 ± 5.65
Body V5% [%]	39	40.43 ± 7.81	39.95 ± 7.70	39.93 ± 7.71	39.85 ± 7.74	39.84 ± 7.81	39.94 ± 7.65	39.96 ± 7.68
PTV High DMax [%]	39	110.1 ± 2.2	110.3 ± 1.7	110.4 ± 1.9	110.1 ± 1.9	110.6 ± 2.3	110.1 ± 2.0	110.4 ± 1.9
PTV High V105% [%]	39	12.5 ± 18.7	16.5 ± 13.6	18.4 ± 14.6	15.1 ± 12.4	16.4 ± 12.5	16.5 ± 14.2	18.2 ± 14.4
PTV Int D95% [cGy]	34	6081 ± 135	6082 ± 137	6085 ± 144	6054 ± 157	6062 ± 156	6087 ± 138	6095 ± 139
PTV Low D95%[cGy]	38	5611 ± 73	5633 ± 81	5636 ± 82	5629 ± 76	5631 ± 74	5648 ± 100	5637 ± 82
Brainstem Dmax [cGy]	38	3278 ± 1265	3194 ± 1203	3198 ± 1192	3160 ± 1173	3170 ± 1195	3180 ± 1192	3179 ± 1192
Cochlea L Dmean [cGy]	39	1179 ± 1325	935 ± 994	968 ± 1074	858 ± 776	580 ± 463	981 ± 1076	877 ± 757
Cochlea R Dmean [cGy]	39	1344 ± 1192	992 ± 872	1027 ± 965	1053 ± 1016	605 ± 359	960 ± 864	988 ± 822
Constrictors Dmean [cGy]	36	4960 ± 1041	4812 ± 1158	4782 ± 1171	4772 ± 1214	4921 ± 1106	4819 ± 1139	4898 ± 1100
Cord Dmax [cGy]	39	3566 ± 636	3594 ± 359	3606 ± 354	3594 ± 351	3548 ± 374	3577 ± 408	3596 ± 361
Eye L Dmax [cGy]	26	823 ± 1243	648 ± 874	664 ± 895	603 ± 756	628 ± 828	689 ± 958	623 ± 828
Eye R Dmax [cGy]	26	886 ± 1390	797 ± 1255	805 ± 1250	794 ± 1229	793 ± 1282	808 ± 1260	815 ± 1265
Larynx Dmean [cGy]	27	4065 ± 1444	3779 ± 1463	3728 ± 1470	3707 ± 1463	3792 ± 1450	3851 ± 1463	3769 ± 1480
Lips Dmean [cGy]	5	1473 ± 497	1614 ± 625	1621 ± 610	1715 ± 651	1750 ± 647	1707 ± 718	1579 ± 575
Mandible Dmax[cGy]	38	6771 ± 964	6777 ± 1079	6806 ± 1085	6748 ± 1119	6786 ± 1085	6809 ± 1072	6780 ± 1075
Optic Chiasm Dmax [cGy]	24	731 ± 1372	680 ± 1312	685 ± 1323	687 ± 1335	681 ± 1331	713 ± 1355	693 ± 1332
Optic Nerve L Dmax [cGy]	22	926 ± 1432	763 ± 1228	753 ± 1222	770 ± 1252	757 ± 1217	755 ± 1216	759 ± 1222
Optic Nerve R Dmax [cGy]	23	963 ± 1642	821 ± 1464	814 ± 1445	826 ± 1461	822 ± 1465	811 ± 1457	814 ± 1456
Oral Cavity Dmean [cGy]	38	3717 ± 1205	3791 ± 1176	3804 ± 1186	3798 ± 1160	3781 ± 1163	3800 ± 1186	3786 ± 1197
Parotid L Dmean [cGy]	37	2211 ± 900	2286 ± 852	2260 ± 830	2282 ± 912	2293 ± 930	2251 ± 840	2276 ± 837
Parotid L V2600cGy [%]	37	32 ± 18	33 ± 17	32 ± 17	33 ± 18	33 ± 18	32 ± 17	33 ± 17
Parotid R Dmean [cGy]	36	2374 ± 844	2419 ± 752	2417 ± 767	2462 ± 825	2479 ± 821	2418 ± 746	2426 ± 760
Parotid R V2600cGy [%]	36	35 ± 15	36 ± 14	36 ± 14	38 ± 17	38 ± 17	36 ± 13	36 ± 14
Submandibular L Dmean [cGy]	29	4245 ± 1961	4307 ± 1792	4286 ± 1951	4441 ± 1797	4494 ± 1674	4309 ± 1780	4199 ± 1909
Submandibular R Dmean [cGy]	20	4178 ± 1569	4232 ± 1570	4219 ± 1486	4254 ± 1557	4262 ± 1486	4120 ± 1473	4100 ± 1590

*Note*: Overview of dosimetric parameters across the entire patient cohort for the clinical plans, and plans re‐planned with *KBP_Full_*, *KBP_101_*, *KBP_50_*, *KBP_25_*, *KBP_A_*, and *KBP_B_*. Values given as mean ± standard deviation across the 39 plans.

**TABLE 4:** Dosimetric differences between plans.

Parameter	*n*	Clinical—*KBP_Full_*	*KBP_Full_*—*KBP_101_*	*KBP_Full_*—*KBP_50_*	*KBP_Full_*—*KBP_25_*	*KBP_A_*—*KBP_B_*
Conformity index	39	0.035 ± 0.053^*^ (*p* < 0.001)	−0.002 ± 0.009	0.005 ± 0.022	0.004 ± 0.025	−0.004 ± 0.012^*^ (*p* = 0.033)
Homogeneity index	39	−0.004 ± 0.023	−0.001 ± 0.004	0.002 ± 0.009	0.001 ± 0.009	−0.001 ± 0.004
Body V100% [%]	39	0.041 ± 0.070^*^ (*p* < 0.001)	−0.003 ± 0.010	0.005 ± 0.019	0.003 ± 0.020	0.016 ± 0.136
Body V50% [%]	39	0.917 ± 0.998^*^ (*p* < 0.001)	−0.025 ± 0.157	0.034 ± 0.243	0.087 ± 0.336	−0.020 ± 0.183
Body V20% [%]	39	0.662 ± 1.007^*^ (*p* < 0.001)	−0.055 ± 0.333	−0.024 ± 0.348	0.036 ± 0.413	−0.063 ± 0.360
Body V5% [%]	39	0.480 ± 0.999^*^ (*p* = 0.004)	0.018 ± 0.213	0.101 ± 0.287^*^ (*p* = 0.035)	0.115 ± 0.389	−0.022 ± 0.257
PTV high DMax [%]	39	−0.2 ± 1.7	−0.1 ± 0.7	0.1 ± 1.0	−0.3 ± 1.6	−0.3 ± 0.8^*^ (*p* = 0.028)
PTV high V105% [%]	39	−4.0 ± 18.0	−1.9 ± 5.1^*^ (*p* = 0.025)	1.3 ± 8.1	0.1 ± 8.1	−1.7 ± 3.9^*^ (*p* = 0.009)
PTV Int D95% [cGy]	34	−1.7 ± 78	−2.5 ± 16.6	3.8 ± 23.5	−4.4 ± 28	−6.3 ± 12.1^*^ (*p* = 0.005)
PTV low D95%[cGy]	38	−22.7 ± 59.8^*^ (*p* = 0.027)	−3.1 ± 10.1	4.3 ± 18.5	2.4 ± 18.5	10.8 ± 98.1
Brainstem Dmax [cGy]	38	83 ± 359	−4 ± 96	35 ± 152	24 ± 158	1 ± 147
Cochlea L Dmean [cGy]	39	244 ± 398^*^ (*p* < 0.001)	−33 ± 94^*^ (*p* = 0.033)	77 ± 255	355 ± 620^*^ (*p* = 0.001)	104 ± 354
Cochlea R Dmean [cGy]	39	352 ± 528^*^ (*p* < 0.001)	−34 ± 127	−61 ± 172^*^ (*p* = 0.034)	388 ± 639^*^ (*p* < 0.001)	−28 ± 105
Constrictors Dmean [cGy]	36	148 ± 281^*^ (*p* = 0.003)	30 ± 97	40 ± 170	−109 ± 184^*^ (*p* = 0.001)	−79 ± 169^*^ (*p* = 0.008)
Cord Dmax [cGy]	39	−27 ± 472^†^ (*p* < 0.001)	−12 ± 158	0 ± 163	45 ± 192	−19 ± 107
Eye L Dmax [cGy]	26	175 ± 462	−16 ± 74	45 ± 171	19 ± 126	66 ± 149^*^ (*p* = 0.034)
Eye R Dmax [cGy]	26	89 ± 276	−8 ± 60	3 ± 62	4 ± 73	−7 ± 52
Larynx Dmean [cGy]	27	286 ± 501^*^ (*p* = 0.006)	51 ± 91^*^ (*p* = 0.007)	72 ± 157^*^ (*p* = 0.025)	−12 ± 254	82 ± 291
Lips Dmean [cGy]	5	−141 ± 181	−7 ± 59	−102 ± 95	−136 ± 78^*^ (*p* = 0.018)	128 ± 144
Mandible Dmax[cGy]	38	−6 ± 200	−29 ± 69^*^ (*p* = 0.014)	28 ± 136	−10 ± 121	29 ± 144
Optic Chiasm Dmax [cGy]	24	52 ± 164	−6 ± 22	−7 ± 36	−2 ± 36	20 ± 56
Optic Nerve L Dmax [cGy]	22	163 ± 412	11 ± 64	−7 ± 55	6 ± 81	−3 ± 69
Optic Nerve R Dmax [cGy]	23	143 ± 538	7 ± 37	−5 ± 31	−1 ± 30	−3 ± 19
Oral Cavity Dmean [cGy]	38	−74 ± 339	−13 ± 54	−6 ± 108	10 ± 130	14 ± 83
Parotid L Dmean [cGy]	37	−74 ± 177^*^ (*p* = 0.015)	26 ± 100	4 ± 252	−7 ± 277	−25 ± 58^*^ (*p* = 0.011)
Parotid L V2600cGy [%]	37	−1 ± 5	1 ± 2	0 ± 5	0 ± 6	−1 ± 2^*^ (*p* = 0.046)
Parotid R Dmean [cGy]	36	−45 ± 206	2 ± 37	−43 ± 326	−60 ± 326	−8 ± 47
Parotid R V2600cGy [%]	36	−1 ± 4	0 ± 1	−2 ± 11	−2 ± 10	0 ± 2
Submandibular L Dmean [cGy]	29	−63 ± 379	22 ± 302	−134 ± 249^*^ (*p* = 0.007)	−187 ± 260^*^ (*p* < 0.001)	110 ± 223^*^ (*p* = 0.013)
SubmandibularR Dmean [cGy]	20	−55 ± 511	13 ± 199	−22 ± 133	−30 ± 178	20 ± 175

*Note*: The table compares the dosimetric parameters between the clinical plans and different KBP plans. Values shown represent the average difference across all 39 patients ± the standard deviation of all (up to) 39 differences. Negative average values thus indicate the former plan cohort having a lower dose value. Asterisk (^*^) and dagger (^†^) highlight significant differences (*p* < 0.05) from paired *t*‐test and *F*‐test, respectively. *p*‐values are given in parentheses when significant.

### 3.1 Overall KBP model quality

**FIGURE 1:**
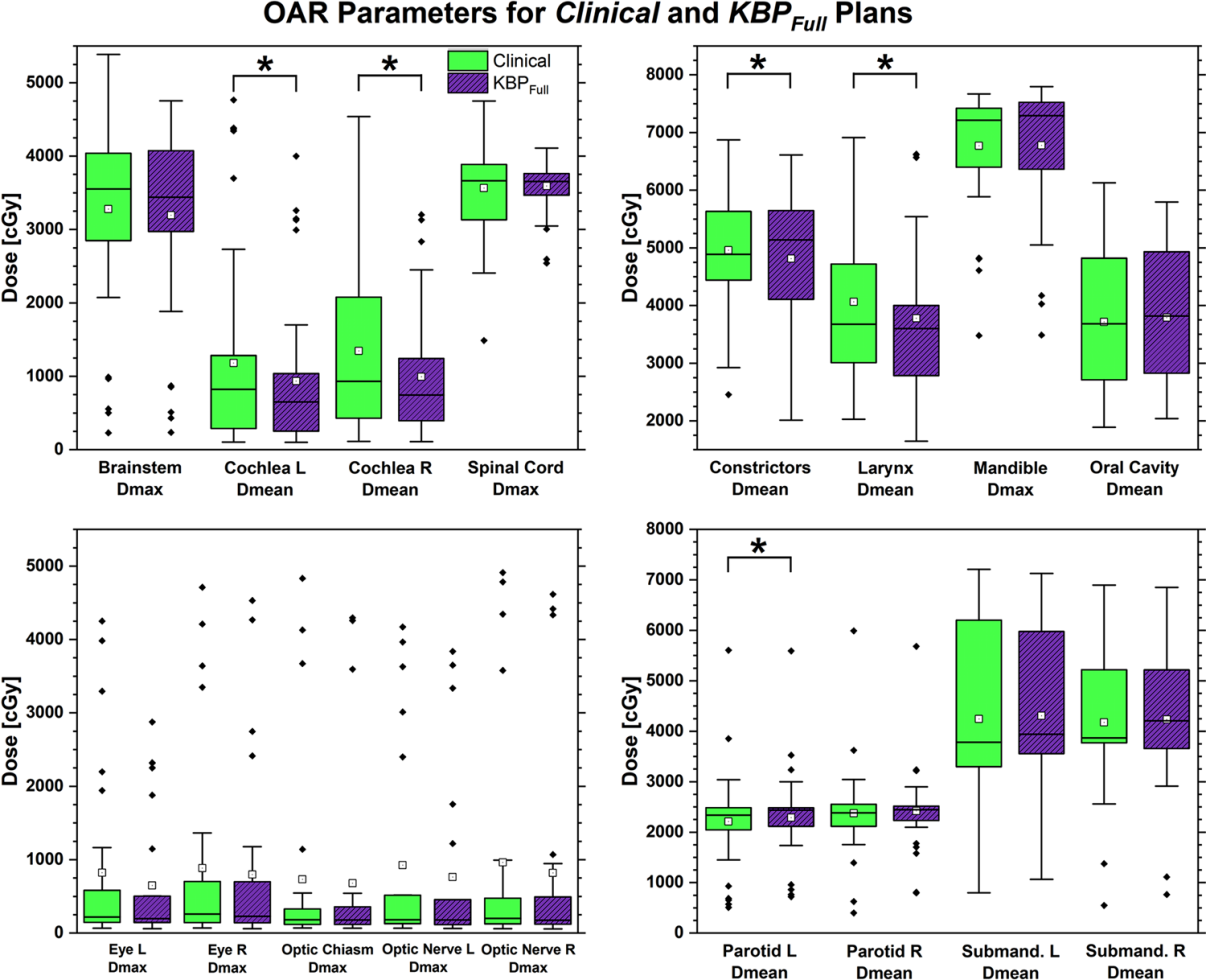
Boxplot of dosimetric parameters for organs‐at‐risk comparing manual clinical plans to plans generated by *KBP_Full_*. Boxes represent quartile groups 2 and 3 separated by the median line for the entire cohort of plans. Whiskers denote minimum/maximum within 1.5 times interquartile range. White squares show average values and black diamonds outliers. Asterisks (^*^) and brackets highlight statistical significance (*p* < 0.05) in paired *t*‐test.

Figure 1 displays the comparison of manual clinical plans and *KBP_Full_. KBP_Full_* significantly increased PTV Low D95% by 23 ± 60 cGy (average ± standard deviation across all 39 patients). The left parotid mean dose was also significantly increased by 74 ± 177 cGy in the *KBP_Full_* plans. While not significant (*p* = 0.176), *KBP_Full_* plans trended towards higher PTV High V105% (16.5 ± 13.6%) compared to the clinical plans (12.5 ± 18.7%). On the other hand, *KBP_Full_* significantly reduced left cochlea mean dose by 244 ± 398 cGy, right cochlea mean dose by 352 ± 528 cGy, constrictors mean dose by 148 ± 281 cGy, and larynx mean dose by 286 ± 501 cGy. Body V100%, V50%, V20%, and V5% were significantly reduced in *KBP_Full_* by 0.04 ± 0.07%, 0.92 ± 1.00%, 0.66 ± 1.01%, and 0.48 ± 1.00%, respectively. The difference in CI between clinical plans (1.090 ± 0.076) and *KBP_Full_* plans (1.054 ± 0.065) was also significant. The *F*‐test showed that the variance in cord Dmax was significantly lower in *KBP_Full_* plans (average of 3594 ± 359 cGy) compared to clinical plans (average of 3566 ± 636 cGy). No other target or OAR parameters showed significant differences.

Overall, the *KBP_Full_* model was demonstrated to produce plans that are at least of similar dosimetric quality to the human planners. Aggregate differences over 39 patients in the analyzed parameters between cohorts ranged from −74 cGy (clinical plans were better) to +352 cGy (*KBP_Full_* plans were better).

The blind review by an independent physician showed that the physician preferred *KBP_Full_* plans over the manual clinical plans in 20/39 cases (51.3%). When *KBP_Full_* plans were preferred, the physician explained the choice with improved OAR sparing in 19/20 cases and a reduced hotspot in 1/20 cases. When the manual clinical plan was chosen, the physician noted OAR sparing in all 19 cases as the reason, with the submandibular glands being most common (11 cases) and parotid glands 2nd most common (three cases). The physician considered 5/39 manually generated clinical and 7/39 *KBP_Full_* plans not acceptable. The lack of OAR sparing was given as a reason for all but one case. Lack of PTV High coverage was given as the reason for the other case (both clinical and *KBP_Full_*).

### 3.2 Effect of KBP model size

**FIGURE 2:**
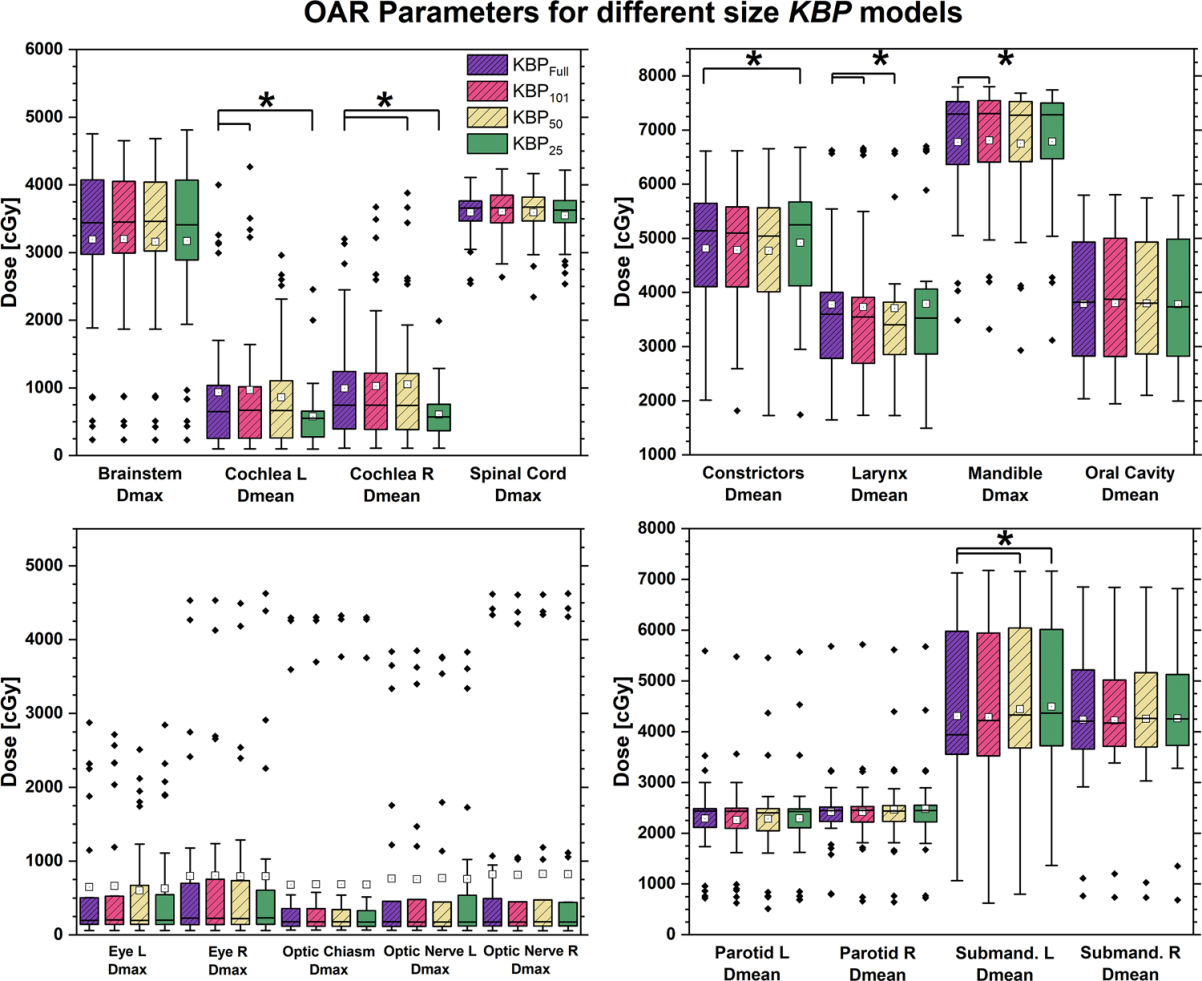
Boxplot of dosimetric parameters for organs‐at‐risk comparing plans generated by *KBP_Full_* to those generated by *KBP_101_*, *KBP_50_*, and *KBP_25_*. Statistically significant differences (*p* < 0.05) in the paired *t*‐test to *KBP_Full_* highlighted with brackets and asterisk (^*^).

The dosimetric comparison of the *KBP_Full_* and *KBP_101_*_,_ *KBP_50_*, *KBP_25_* models is illustrated in Figure 2. The significant PTV High V105% difference of −1.9 ± 5.1% indicates the PTV High was on average slightly hotter in the *KBP_101_* model. For OARs, left cochlea mean dose and mandible max dose were significantly increased in the *KBP_101_* model by 34 ± 127 cGy and 29 ± 69 cGy, respectively. Mean larynx dose was reduced by 51 ± 91 cGy in the *KBP_101_* model. No other differences were statistically significant. With aggregate differences ranging from −34 cGy to +51 cGy, differences between *KBP_Full_* and *KBP_101_* seem minimal.

Compared to *KBP_Full,_* the *KBP_50_* plans significantly increased right cochlea Dmean by 61 ± 172 cGy and left submandibular Dmean by 134 ± 249 cGy, but significantly decreased larynx Dmean by 72 ± 157 cGy. No other changes were significant.

Several OAR doses were significantly different between *KBP_Full_* and *KBP_25_*. *KBP_25_* plans significantly increased Dmean to constrictors, left submandibular, and lips by 109 ± 184, 187 ± 260, and 136 ± 78 cGy, respectively. Surprisingly, Dmean for left and right cochlea was significantly reduced by 355 ± 620 and 388 ± 639 cGy, respectively, in *KBP_25_*.

Overall, it appears that training size differences between the different KBP models resulted in only small differences between plans when compared to the prescription dose. No model appeared to be clearly better or worse than the others. The cochlea in the *KBP_25_* model represent a notable exception.

### 3.3 Effect of physician preferences

**FIGURE 3:**
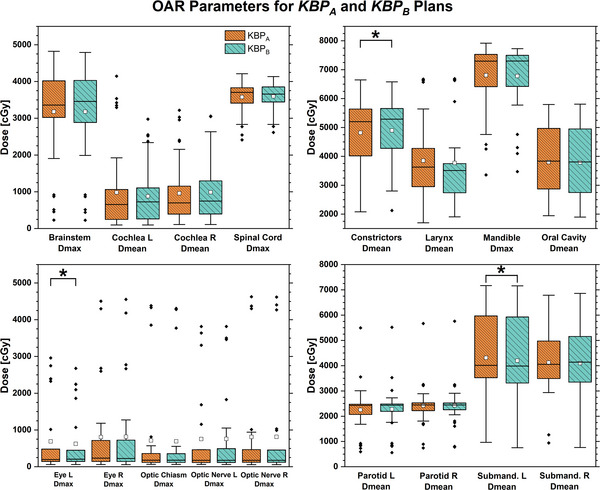
Boxplot of dosimetric parameters for organs‐at‐risk comparing plans generated by *KBP_A_* to those generated by *KBP_B_*. Statistically significant differences in paired *t*‐test shown with asterisks (^*^) and brackets (*p* < 0.05).

Figure 3 depicts the comparison of the OARs in *KBP_A_* and *KBP_B_* models. The *KBP_A_* model showed significantly reduced PTV High Dmax (−0.3 ± 0.8%) and V105% (−1.7 ± 3.9%), PTV Int D95% (−6.3 ± 12.1 cGy), constrictors mean dose (−79 ± 169 cGy), and left parotid mean dose (−25 ± 58 cGy) compared to *KBP_B_*. *KBP_B_* significantly reduced left eye max dose by 66 ± 149 cGy and left submandibular mean dose by 110 ± 223 cGy. Other differences were not significant. Aggregate differences between *KBP_A_* and *KBP_B_* ranged from −79 to +110 cGy and were thus relatively small compared to the target dose of 6000−7000 cGy.

## 4 DISCUSSION

In this work, six KBP models were trained for head‐and‐neck to explore multiple questions. Models were trained with varied model sizes and plan compositions and then used to re‐plan a set of 39 test plans. Optimization objectives were identical except for the line objectives which are dependent on the training set. Plans resulting from the KBP models were compared to clinical manually generated plans and each other.

### 4.1 Overall KBP model quality

The dosimetric comparison of clinical and *KBP_Full_* plans showed KBP was able to significantly reduce the dose to cochlea, constrictors, and larynx mean dose. On the other hand, *KBP_Full_* increased left parotid mean dose by 74 ± 177 cGy on average. These results implied that KBP was able to match clinical plans dosimetrically but has the potential for further improvement with a refined model.

A reduction in the variability of the cord max dose in *KBP_Full_* plans was the only significant difference when looking at planning variability. In this study, variability for a DVH parameter was evaluated over the entire patient cohort and may thus be dominated by variability across patients. The variability among human planners for the same plan could not be assessed as part of this study.

The blind review of clinical and *KBP_Full_* plans showed the physician preferred KBP plans in 20/39 cases. The physician deemed 7/39 *KBP_Full_* plans unacceptable compared to 5/39 manual plans. It was suggested to improve the parotid and/or submandibular dose in the cases where the manual plan was preferred or *KBP_Full_* plans were deemed unacceptable. This suggestion is in line with the dosimetric results that showed a small but significant increase of left parotid dose in *KBP_Full_* plans. Plans from this model also appear to be slightly hotter (in terms of V105%) than the clinical plans, representing another issue that could be addressed. Of note, the *KBP_Full_* plans represent the direct output without any human intervention. In a clinical environment, a human planner could refine the initial KBP output to improve specific DVH constraints.

Nevertheless, future iterations of our *KBP_Full_* HN model will aim at improving parotid and submandibular dose as well as hot spots. This could be achieved, for example, by increasing priorities on the existing objectives, adding optimization structures for parts that do not overlap targets, and changing the model to account for ipsilateral and contralateral OARs rather than left and right location. Further, retraining the KBP model with KBP generated plans could potentially enhance resulting plan quality as has been demonstrated in previous literature.^29^ Despite the potential for improvement, the dosimetric results and blinded physician review indicate the model is suitable for clinical application and produces plans that are at least of a similar quality to plans that were manually generated in previous treatments.

### 4.2 Effect of KBP model size

The effect of model size was evaluated by comparing the plans generated by the *KBP_Full_* model, consisting of 203 training plans, to models trained with 101, 50, and 25 plans. Average differences to *KBP_Full_* were at most 51, 134, and 187 cGy for the *KBP_101_*, *KBP_50_*, and *KBP_25_* models, respectively. A notable exception was observed in the *KBP_25_* model where average cochlea mean dose across the 39‐test patient cohort was reduced by up to 388 cGy. This is an unexpected result given the general assumption that increasing the number of training plans will result in better KBP generated plans. This behavior may be a consequence of only using line objectives for the cochlea in our models. We noticed two effects at play in the *KBP_25_* model. The DVH estimation was lower than in the *KBP_Full_* model, indicating this particular subset of the training cohort had a lower cochlea dose. Secondly, the standard deviation of the DVH estimation was also larger. In the chosen KBP solution, both of these lead to the generation of a stricter line objective and thus lower cochlea dose in the end. Despite already producing a significantly lower cochlea dose than the clinical plans, this indicates the *KBP_Full_* model could be further improved for this OAR.

Besides the cochlea, aggregate dose differences across the different model sizes were small compared to the prescription dose. We therefore conclude that KBP model size did not have a substantial impact on the resulting plan quality in terms of dosimetric parameters. An important addendum to this conclusion is that KBP users need to understand the minimum requirements for training their KBP models. For example, the KBP solution chosen for this study needs at least 20 instances of an OAR to be able to create DVH estimations. Without this minimum number, no DVH estimations can be created likely leading to an increase in OAR dose for KBP plans.

The results from the different size KBP models confirm the findings of another published study that investigated the effects of model size in KBP for the prostate with models comprising 31, 66, and 97 patients.^26^ From the results in this present and the previous study, it seems a prudent conclusion that clinics looking to employ KBP can start by training a small model and adding on to the model over time without having to expect a drastic change in resulting plans after retraining.

### 4.3 Effect of physician preferences

Finally, the dosimetric comparison of KBP models generated by plans from two different physicians also showed minimal differences. Despite differences of 500−1500 cGy in prescribed OAR dose, KBP plans had aggregate differences of 110 cGy or less. As a limitation, only two physicians could be compared in this study. Further, the plans in the training sets were not fully independent of each other because of an overlap of planners. Future studies could include more physicians and categorize models additionally by the planner. Finally, full physician review of the plans generated by the different KBP models would strengthen the analysis but was beyond the scope of this study.

As it stands, however, the results are interpreted as an argument for training larger KBP models that encompass a variety of patients, physicians, and planners rather than creating specific models for each physician and planner. As an added benefit, this reduces the effort needed for maintenance and continued improvement of KBP models in the clinic. If physicians have differing opinions on clinical tradeoffs, these could manually be incorporated by the planner as optimization objectives rather than needing to undergo the arduous task of training and testing separate KBP models. Further, this also provides evidence that clinics lacking a sufficiently large database of previous plans could import a KBP model from an outside source and adjust optimization objectives according to their planning philosophy.

## 5 CONCLUSIONS

Several head‐and‐neck KBP models were developed and compared. The analysis showed training models with different head‐and‐neck planning databases yielded only small dosimetric differences in resulting KBP plans. Therefore, clinics looking to implement KBP are encouraged to train models that do not separate between different planners or physician preferences. Alternatively, KBP models can be imported from an outside source. Using adequate optimization objectives these models will be able to generate plans suited for each clinic's demands. As more planning data becomes available over time, these models can be retrained using the larger database, without concerns of drastic changes in resulting plan quality. These findings reduce the burden of the initial roll‐out of KBP into the clinic.

## AUTHOR CONTRIBUTIONS

All authors made substantial contributions to conception of the work, analysis, interpretation, drafting the work and gave final approval of the manuscript.

## CONFLICT OF INTEREST STATEMENT

The authors declare no conflicts of interest.

## Data Availability

The data that support the findings of this study are available on request from the corresponding author. The data are not publicly available due to privacy or ethical restrictions.
